# Supplementary material for: ProteinShader: illustrative rendering of macromolecules
Source: BMC Struct Biol. 2009 Mar 30;9:19. doi: 10.1186/1472-6807-9-19 (PMC2672931; doi:10.1186/1472-6807-9-19)
Supplement: Additional file 1 — ProteinShader program without source code. This compressed file contains the complete ProteinShader program including associated libraries, but no source code. A README.txt file gives an overview of the ProteinShader distribution, and the index.html file in the help subdirectory has directions on getting started with the program as well as a set of tutorials. [file 1472-6807-9-19-S1.zip › ProteinShader-beta-0_9_4-binary/help/tutorials/2BNH.html]

ProteinShader Tutorial: 2BNH


# ProteinShader Tutorial


♦ Home


♦ Download


♦ Getting Started

Choose a Link:

Installation

Running

Troubleshooting

PDB Files


♦ Tutorials

Choose a Link:

List of Proteins

Style Options

Saving Images


♦ General Controls

Choose a Link:

Menu Bar

Keyboard Shortcuts

Mouse Motion

Motion Panel

Antialiasing


♦ Cartoon Controls

Choose a Link:

Control Panels

Selection Panel

Radio Buttons

Decorations Panel

Adding Textures

Color Panel

Visibility Panel

Side Chains Panel


♦ Atom Controls

Choose a Link:

Control Panels

Selection Panel

Radio Buttons

Color Panel

Visibility Panel

Scale Panel

Tiling Panel


♦ Developers

Choose a Link:

Compiling

Running

Code Docs

Developer Links


## Ribonuclease Inhibitor: Alpha/Beta Horseshoe-Shaped Structure [PDB:2BNH]

The ribonuclease inhibitor protein is a horseshoe-shaped structure
that is built from a repeating leucine-rich motif that corresponds
to a beta-strand/loop/alpha-helix unit [1,
2, 3,
4].
These units are arranged so that the inside part of the horseshoe contains
a parallel beta-sheet, while alpha-helices form the outside part.
The three-dimensional structure of the porcine ribonuclease inhibitor has been
solved by x-ray crystallography
[2, 3],
and can be loaded by selecting Open from the
File menu
of the ProteinShader program and then using the file chooser box to select the
2BNH.pdb file.


## Cartoon-style displays with halftoning

When the ribonuclease inhibitor is first loaded, it will be displayed as a
pen-and-ink style drawing of ribbons and tubes as shown in Figure 1A. In the
default orientation, the beta-strands (wide ribbons) are mostly obscured by the
alpha-helices (spiral tubes), but the mouse can be dragged across the canvas to
rotate the image and get a better sense of the protein's three-dimensional
structure. The protein can be returned to its original orientation at any
time by going to the
Orientation menu
menu above the canvas and selecting Original.

To switch from the Figure 1A image to the Figure 1B image, first drag the
mouse horizontally or vertically across the canvas to get a side view of
the horseshoe shape. Then go to the
Decorations subpanel
of the Control Panel on the right side of the canvas, and click on the
β-Strands radio button to select All beta-strands. Next, use the Halftone
Texture menu right below the Halftoning button to select a Vertical Bars
pattern to map onto the surface of the beta-strand ribbons, and
set the Bend Texture menu to None (the bend texture is applied to the
middle of each segment of the ribbon that corresponds to an amino acid,
and the intensity of the bend texture is scaled according to how curved the
ribbon or tube segment is).

To fade out the loop regions that connect the beta-stands, go to the menu at
the top right of the Control Panel and use it to change from the
Decorations subpanel
to the 
Cartoon Visibility subpanel. Click on the Loops radio button to select
all loops, and then click on the Translucent button. By default, the button
should be set to use 75% translucency, but the value can be changed by using
the slider control below the button.


|  |  |
| --- | --- |
| **A** | **B** |

**Figure 1.** The ribonuclease inhibitor protein is a horseshoe-shaped
structure.
(A) A ribbons and tubes display of this single-chain protein using
halftoning to produce a pen-and-ink style drawing.
(B) The protein has been rotated to a view that makes the horseshoe shape more
obvious. Beta-strands are shown as ribbons with stripes, and they form the
inner part of the horseshoe shaped structure, while alpha-helices are shown as
tubes, and they form the outer part of the horseshoe. The loop regions are
shown as semi-transparent thin ribbons.
The images were captured as described in the
Saving Images
section of the tutorials main page.


## Cartoon-style displays with color

The 
Decorations subpanel
can also be used to select color images for ribbons and tubes, as seen in Figure
2. To set the beta-strands to a color image with a pattern mapped onto the
surface of the ribbons, first select the β-Strands radio button, and then
click on the Patterns button. The menu right below the Patterns button is set
to Vertical Bars by default, but can be changed to any of several other images.
The default region color for beta-strands is green, but they can be set to any
color (or colored by amino acid type) by using the
Cartoon Color subpanel.

To change the alpha-helices to match Figure 2, select the Helices button near
the top of the Decorations subpanel, and then click the Plain button at the
very bottom of the panel (plain here means color images with no special
patterns mapped onto the surfaces of the ribbons and tubes). The default
region color for alpha-helices is red, but as with beta-strands and loops, the
Cartoon Color subpanel
can be used to set them to any color, and the background color of the canvas can
be changed to black by using the
Background menu
above the canvas.


|  |  |
| --- | --- |
| **A** | **B** |

**Figure 2.** Color images of the ribonuclease inhibitor protein. (A) The
protein is presented in the same view as Figure 1B, but with beta-strands shown
as green ribbons with black stripes and alpha-helices as red tubes. Specular
highlighting (the shiny plastic-like appearance) is used to enhance the
three-dimensionality of the images. (B) The image in part A has been rotated
so that the open end of the horseshoe is closest to the viewer. For tubes and
ribbons shown in color, a blue end cap marks the amino-terminus, while
a red end cap marks the carboxyl terminus.
The images were captured as described in the
Saving Images
section of the tutorials main page.


## References

1.
Brandon C, Tooze J: **Alpha/Beta Structures.**
In *Introduction to Protein Structure.* 2nd edition.
New York: Garland Publishing; 1998: 47-65.

2.
Kobe B, Deisenhofer J:
**Mechanism of ribonuclease inhibition by ribonuclease inhibitor protein
based on the crystal structure of its complex with ribonuclease A.**
*J Mol Biol* 1996, **264**: 1028-1043.

3.
**Ribonuclease inhibitor protein PDB entry 2BNH**
[http://www.rcsb.org/pdb/explore/explore.do?structureId=2BNH]

4.
Kobe B, Kajava AV:
**The leucine-rich repeat as a protein recognition motif.**
*Curr Opin Struct Biol* 2001, **11**: 725-732.


---

|  |  |  |  |
| --- | --- | --- | --- |
| Hosted by |  |  |  |

Webmaster: Joe Weber,
joe.weber AT alumni.duke.edu
  
Web site last updated: December 30th, 2008
  
© 2007–2008 Joseph R. Weber
